# Predictors for Using a HIV Self-Test Among Migrant and Seasonal Farmworkers in North Carolina

**DOI:** 10.3390/ijerph120708348

**Published:** 2015-07-17

**Authors:** Samantha Kinney, C. Suzanne Lea, Greg Kearney, Anna Kinsey, Carlos Amaya

**Affiliations:** 1 Department of Health and Human Services, Mendocino County Health and Human Services Agency, Public Health Services, Ukiah, CA 95482, USA; E-Mail: kinneys@co.mendocino.ca.us; 2 Department of Public Health, Brody School of Medicine, East Carolina University, Greenville, NC 27834, USA; E-Mail: kearneyg@ecu.edu; 3 Kinston Community Health Center, 324 N Queen St., Kinston, NC 28501, USA; E-Mails: akinsey@kinstonhealth.org (A.K.); camaya@kinstonhealth.org (C.A.)

**Keywords:** HIV testing, seasonal and migrant farmworkers, behavioral factors, survey, North Carolina

## Abstract

*Background:* Approximately, two million migrant and seasonal farmworkers (MSF) work in the United States annually. Several factors, such as lack of access to healthcare services and health behaviors, contribute to risk of HIV transmission. Relatively few studies have explored MSF knowledge of HIV transmission and testing options. *Methods:* A 12-question, self-administered survey of farmworkers (*n* = 178) from 19 migrant camps was conducted. The survey assessed knowledge of factors related to HIV transmission, testing, and intention to use a HIV home-test kit. *Results:* Participants with knowledge of treatment for HIV (*p* = 0.03) and that condom use protects against HIV (*p* = 0.04) were more willing to express intent to use a home test kit than those with less knowledge. Concern among farmworkers that HIV was a very or somewhat serious problem in their community was associated with expressing intent to use a home test kit (OR = 2.3, 95% CI = 0.92–5.5). Respondents with less knowledge were less likely to use a home test kit. *Conclusions*: MSF were concerned about HIV in their community and would be willing to use to a home test kit. This pilot study provides a basis for additional research related to HIV testing within the MSF community.

## 1. Introduction

Hispanic and Latinos are disproportionately affected by new HIV infections. Nationally, in 2010, the rate of new HIV infections for Latino males was 2.9 times that for white males [1]. In 2013 in North Carolina, Hispanic males (ages 13+) had a rate of new HIV diagnosis that was 2.7 times greater than white non-Hispanic males, 32.5 per 100,000 and 12.0 per 100,000, respectively [2]. While national data suggest that increasing HIV rates in Hispanic men are due to men having sex with men, these data are primarily reported from large urban populations [3]. Hispanic migrants entering the U.S. from Mexico and Latin America in search of work may contribute to HIV transmission through increased risky behavior [4]. Increasing access to testing, while promoting risk reduction, are important public health measures to decrease the number of diagnoses of HIV infection among disproportionately affected population groups [3].

Migrant and seasonal farmworkers (MSF), most of whom are male and originate from Mexico and Central America, represent a vulnerable population at risk for undiagnosed HIV. Few recent studies have characterized the incidence rate or prevalence of HIV infection among farmworkers [5,6,7]. Among the estimated 35,000 migrant workers employed in the agriculture industry in North Carolina annually, the majority are poor, lack access to healthcare, and do not speak English [6]. Within this context, many factors contribute to the increasing susceptibility of risk of HIV among farmworkers, including family separation [8,9], paying a woman to have sex [7,10], binge drinking [7,10], lack of condom use [11,12,13], prevalent sexually transmitted infections (STIs) [7,14], cultural barriers, and limited access to healthcare services [6,8,15]. In addition, social stigmas associated with HIV transmission may influence a farmworker’s choice to decline HIV testing using public health services [14].

In 2012, the U.S. Food and Drug Administration (FDA) approved an over-the-counter, rapid home-use HIV test kit, which detects antibodies in saliva [16,17]. Since one-third of HIV transmission is in the undiagnosed [18], a self-test for HIV may be an effective approach to screen and diagnosis infection in rural, vulnerable farmworkers. Increasing awareness of HIV risk in the MSF by promoting self-testing may be an additional prevention strategy for this population [19]. Few data exist examining knowledge and behaviors related to HIV testing using a HIV home test kit within the Latino MSF community [20,21]. A higher perceived risk of HIV infection may be associated with intention to use a home-based HIV test kit. The purpose of this pilot study was to examine the knowledge and perception of using a HIV home test kit among a sample of MSF over one agricultural growing season in eastern North Carolina.

## 2. Experimental Section

### 2.1. Participants

Participants (*n* = 199) were recruited between April and June 2013 from farmworker labor camps in eastern North Carolina. Access to the study population was provided by a local outreach worker employed by a community health center. The center had a prior program offering free HIV testing. Inclusion criteria for this study included Latino ethnicity and ≥18 years of age. There were no women farmworkers encountered, therefore, the study population was comprised of 100% males. All participants provided oral consent prior to participation. Upon completion of the survey, two condoms were distributed to participants.

### 2.2. Data Collection

Eighteen H-2A camps and 1 non-H-2A camp were visited. H-2A classification is one of many temporary, nonimmigrant worker classifications defined by the U.S. government. Workers residing at H-2A camps are defined as temporary, legal agricultural employees contracted with a farmer to provide services to his/her business [22]. A trained, local outreach worker, a male, native Spanish speaker, identified the dates and locations to conduct the survey. After arriving at the location, the outreach worker would informally explain the purpose, incentive and ask MSF about interest in completing the survey, typically in a group setting. Interested workers completed the survey. When literacy was an issue, the outreach worker would read questions and answer options aloud. After the survey was returned, respondents were offered two free condoms. No personal identifiers were collected. Data collection was conducted in the evening hours, typically after the worker had returned from the fields. The East Carolina University IRB approved this study prior to data collection.

The questionnaire was developed in English and then translated by a qualified Spanish speaking native Mexican. The questionnaire included 12 questions related to sexual behavior, concerns about HIV, sexuality and HIV knowledge. The survey was adapted from the 2000 National Survey of Teens on HIV/AIDS, Public Knowledge and Attitude about HIV/AIDS, sponsored by the Kaiser Family Foundation [23]. Our survey questions were adapted from this national survey because the questions were developed for individuals with middle-school to high-school education, similar to the MSF population. In addition, the survey was designed to be short and simple, given that our visit was unannounced to the workers.

### 2.3. Measures

The primary outcome for this study was assessing whether access to a HIV home test kit would increase HIV testing (“yes”, “no”, “maybe”, “don’t know”): “If you have access to a home HIV testing kit, would you increase your HIV testing?” This was operationalized as intention to use a home test kit [20]. Each question, except the one on HIV testing status, had an option to characterize the absence of knowledge or behavior, such as “Don’t Know” or “Never”, as this information was considered important for future education and outreach efforts.

A composite score was developed for five HIV knowledge questions [20]. Each answer selected from “yes”, “no”, or “don’t know” was equal to 1, 2 or 3 points, respectively, with range of 5 to 15 points possible in the HIV knowledge section. HIV knowledge questions were: (1) “Are there drugs to treat HIV?” (2) “Is there a cure for HIV?” (3) “Can you become infected with HIV by having unprotected oral sex?” (4) “Can sex without a condom increase a person’s risk of becoming infected with HIV?” (5) “Can needle sharing increase a person’s risk of becoming infected with HIV?” Respondents with 1–5 points, 6–10, and 11–15 points, were categorized with sufficient knowledge, some knowledge, and little knowledge, respectively, in terms of HIV education and knowledge.

Likert scale questions included several related to behavior and beliefs. Sexual behavior was measured by “In general, how often do you use a condom?” with response options “all the time”, “most of the time”, “only sometimes”, and “never”. A question regarding concern about HIV was “Is HIV infection a serious problem for seasonal farmworkers?” with response options “very serious”, “somewhat serious”, “not serious” and “don’t know.” Belief was measured by “How likely are you to contract HIV?” with response options, “very likely”, “somewhat likely”, “not at all”, and “don’t know.”

### 2.4. Analysis

Descriptive analysis, including two-way classification by primary outcome, were provided with Chi-square statistics. For logistic regression, “no” and “don’t know” responses were collapsed into “no” for independent and dependent variables. Odds ratios and 95% confidence intervals (CI) were computed using two logistic regression models. Records with missing data for either response (*n* = 6) or predictor variables included in the model were dropped from the logistic regression procedure, leaving 149 out of 178 records to generate odds ratios. All variables from the survey were included in a logistic model to determine odds ratios after adjusting for each variable, respectively. Then to determine the best-fitting model predictors were included and backward elimination of variables (*p* = 0.20 and lower) was used to identify significant predictors. Analysis was conducted using Stata, Version 12 (Stata Corp LP, College Station, TX, USA).

## 3. Results

Of 199 surveys distributed at 19 camps (18 H2A and one non-H2A camp) between 11 April 2013 through 12 June 2013, 178 surveys were returned for an overall response rate of 89%. The lowest response rate for an individual question used in this analysis was 93% (“Can you become infected with HIV by having unprotected sex?”) and the highest response rate was 98% (“Is HIV infection a serious problem for seasonal workers?”).

Table 1 summarizes the survey results. The participants had a high degree of knowledge that condom use protected against HIV and sharing needles increased HIV risk. A majority of MSF (61%) had been tested for HIV in the past. A large majority responded that HIV was a somewhat or a very serious concern in the farmworker community (81%), and used condoms all the time (43%). A majority of farmworkers indicated they would use a home HIV testing kit (59%) and would be more inclined to use the kit if trained on how to use it (45%). Health clinics and providers (72%) were the dominate information source for health education material.

Table 2 presents the intention to use home test kit by knowledge and behaviors. The knowledge composite variable (*p* = 0.03) and history of prior HIV test (*p* = 0.04) were significantly associated with the intent to use home test kits. However, there was no difference in intention to use a home test kit by condom use, concern for HIV in community, or likelihood to contract HIV.

Many respondents, almost 20% on some questions, replied maybe or don’t know to increasing HIV testing if provided access to a home test kit, particularly of interest, given the concern for HIV (81 serious or very serious) in the seasonal farmworker community and 40% of workers only sometimes or never using a condom.

**Table 1:** Knowledge and beliefs of HIV disease among 178 Latino male farmworkers, North Carolina.

Concept	Question	Response Rate	Response Options			
		*n* (%)	Yes	No	Don’t know	Maybe
Access	If you had access to a HIV home testing kit, would you increase your HIV testing?	172 (97)	101 (59)	13 (8)	43 (25)	15 (9)
Knowledge	Are there drugs to treat HIV?	171 (96)	36 (21)	64 (37)	71 (41)	
	Is there a cure for the HIV virus?	171 (96)	21 (12)	100 (58)	50 (29)	
	Infected by having unprotected oral sex?	166 (93)	97 (58)	21 (13)	48 (29)	
	Sex without condom increases HIV risk?	172 (97)	163 (95)	3 (2)	6 (3)	
	Sharing needle increases HIV risk?	173 (97)	153 (88)	4 (2)	16 (9)	
Test History	Prior HIV test		Yes	No		
		173 (97)	105 (61)	68 (39)		
HIV Concern	Is HIV infection a serious problem for seasonal farmworkers?		Very Serious	Somewhat	Not Serious	Don’t Know
		174 (98)	95 (55)	46 (26)	4 (2)	29 (17)
Sexual Behavior	In general, how often do you use a condom?		All the time	Most of time	Sometimes	Never
		171 (96)	74 (43)	27 (16)	53 (30)	17 (10)
Belief	How likely are you to contract HIV?		Very likely	Somewhat	Not at all	Don't know
		172 (97)	35 (20)	45 (26)	47 (27)	45 (26)
Information	Sources	172 (97)				
	Health Clinic & Providers		128 (71)			
	Friends & Family		10 (6)			
	Internet & T.V.		11 (56)			
	Other		8 (5)			
	No Where		20 (11)			

**Table 2:** HIV Knowledge, attitudes, and beliefs by Intention to use a HIV home-test kit among Latino farmworkers, North Carolina.

Survey Questions	Intention to Use HIV Home Test Kit ^a,b^				
	Yes	No	Maybe	Don’t Know	*p*-value ^c^
	*n* (%)	*n* (%)	*n* (%)	*n* (%)	
HIV Education & Knowledge					
Composite measures					
Little Knowledge **^d^**	11 (55)	1 (5)	3 (15)	5 (25)	
Some Knowledge	71 (60)	9 (8)	33 (28)	6 (5)	
Sufficient Knowledge	10 (71)	2 (14)	2 (14)	0	0.03
Correct responses for components of education and knowledge composite measure **^b^**					
Individual Component / correct response					
Are there drugs to treat HIV? yes	21 (58)	4 (11)	11 (31)	0	0.03
Is there cure for HIV? no	61(62)	9 (9)	24 (25)	4(4)	0.17
Infected by unprotected oral sex? yes	60 (63)	9 (9)	21 (22)	6 (6)	0.43
Sex without condom increases risk? yes	96 (60)	12 (8)	39 (25)	12(8)	<0.01
Sharing needles increases risk? yes	94 (62)	12 (8)	34 (23)	10 (7)	0.06
Concern that HIV is serious problem for seasonal farmworkers					
Very Serious	60 (65)	8 (9)	18 (20)	6 (6)	
Somewhat Serious	29 (64)	3 (7)	10 (22)	3 (7)	
Not Serious	3 (75)	0	1 (25)	0	
Don’t Know	9 (31)	2 (7)	12 (41)	6 (21)	0.08
Sexual behavior, How often do you use a condom?					
All of the time	46 (64)	6 (8)	16 (22)	4 (6)	
Most of the time	13 (48)	2 (7)	9 (33)	3 (11)	
Only sometimes	33 (63)	3 (6)	12 (23)	5 (8)	
Never	7 (44)	1 (6)	5 (31)	3 (19)	0.72
HIV test history					
Yes	65 (63)	6 (6)	23 (22)	9 (9)	
No	35 (53)	7 (11)	19 (29)	5 (8)	0.04
Belief, How likelihood are you to contract HIV?					
Very Likely	23 (66)	4 (11)	7 (20)	1 (3)	
Somewhat Likely	27 (64)	1 (2)	12 (29)	2(5)	
Not at all	28 (60)	3 (6)	11 (23)	5 (11)	
Don't Know	20 (45)	4 (9)	13 (30)	7 (16)	0.34
Health information sources					
Health Clinic & Providers	76 (44)	11 (6)	31 (18)	8 (5)	
Friends & Family	5 (3)	0	1 (1)	3 (2)	
Internet & T.V.	4 (2)	0	5 (3)	1 (1)	
Other	3 (2)	0	2 (1)	2 (1)	
No Where	10 (6)	2 (1)	4 (2)	2 (1)	

**^a^** Question read, “If you had access to a home HIV testing kit, would you increase your HIV testing?”;**^b^** Numbers do not sum to 100 due to missing values and rounding; **^c^** Chi-square test; **^d^** Composite score based on tertiles of knowledge from 1–15, with score 1–5 as high knowledge.

Table 3 presents odds ratios for association between predictors and intention to use home test kit (yes/no). While in univariate analysis, respondents considered HIV a very or somewhat serious concern in seasonal farmworkers (OR = 3.2, 95% CI 1.5–7.2), after adjustment for composite knowledge measure, test history, and likelihood to contract HIV, the association with serious concern and intention to use a test kit was no longer significant (OR = 2.3, 95% CI 0.92–5.5). Both HIV test history and belief to contract HIV were elevated in the adjusted model, although not significantly. Beginning with a full model (education composite, seriousness, condom use, testing history, and belief), each predictor was removed leaving “seriousness” (concern that HIV is a serious or somewhat serious concern in seasonal farmworkers) as the only predictor for intention to use a home test kit in the model (OR = 2.7, 95% CI 1.1–6.2) (data not shown in table). When including each component of knowledge (instead of the composite) and other predictors in backward elimination, “seriousness” emerged as the only predictor significantly associated with intention to use a home test kit (OR = 2.5, 95% CI 1.1–5.8).

**Table 3:** Association between HIV knowledge, attitudes, and beliefs and intention to use a HIV home-test kit among Latino farmworkers, North Carolina.

Survey Questions	Intention to Use HIV Home Test Kit		Odds Ratio (95% Confidence Interval)
	Yes	No ^a^	Adjusted ^b^
	*n*	*n*	
HIV Education & Knowledge			
Composite measures			
Little Knowledge **^c^**	11	9	1.0
Some Knowledge	71	48	1.0 (0.38–2.9)
Sufficient Knowledge	10	4	1.5 (0.31–6.7)
Individual Component / correct response			
Are there drugs to treat HIV? yes	21	15	0.92 (0.44–1.9) **^d^**
Is there cure for HIV? no	61	37	1.4 (0.75–2.6)
Infected by unprotected oral sex? yes	60	36	1.5 (0.79–2.8)
Sex without condom increases risk? yes	96	63	7.5 (0.85–65.3)
Sharing needles increases risk? yes	94	56	2.8 (1.07–7.7)
Concern that HIV is serious problem for seasonal farmworkers			
Not Serious/Don’t know	12	21	1.0
Very/somewhat Serious	89	48	2.3 (0.92–5.5)
Sexual behavior, condom use			
Sometimes/never	40	28	1.0
All/most of time	59	40	1.0 (0.5-1.9) **^c^**
HIV test history			
No	35	32	1.0
Yes	65	38	1.6 (0.80–3.2)
Belief, likelihood to contract HIV			
Not at all/Don’t Know	48	43	1.0
Very/somewhat Likely	50	27	1.4 (0.64–2.6)

**^a^** “No” category includes respondents with no, maybe and don't know; **^b^** Model includes composite knowledge measure, concern for HIV, test history, and likelihood to contract HIV; **^c^** Composite score based on tertiles of knowledge from 1–15, with score 1–5 as high knowledge; **^d^** Individual components of knowledge and education composite variable with univariate analysis.

## 4. Discussion

The results of this study indicate that MSF consider HIV a serious concern in their community and would be motivated to use a home test kit to identify HIV status. Workers with sufficient knowledge of HIV risk factors or had been previously tested were more likely to express intent to use a test kit than those who were not as knowledgeable or had not been tested. Many farmworkers had prior HIV testing, which was also a motivator to use a HIV test kit. Those with little knowledge or prior HIV testing were least likely to intend to use a HIV home test kit.

Because HIV home test kits are relatively new, few studies were found investigating HIV testing behaviors in MSF and their intention to use a HIV home test kit. In a South Florida population of Hispanic farmworkers, Fernandez and colleagues surveyed intention to accept a free HIV test. Our findings were similar to Fernandez [20] and colleagues who found that farmworkers having been tested for HIV have intent to accept a free HIV test. In addition, “worrying” some or a lot about getting HIV indicated testing, similar to our measure of likelihood of “contracting” HIV and using a home test kit. While Fernandez did not have a measure of HIV in the community of farmworkers, our measure of community concern (that HIV is a serious concern in the farmworker community) may indicate some individual-level of “worrying”. Unprotected sex was not a strong predictor for test use [20]. Our findings may be consistent with Fernandez in that HIV knowledge was a predictor of intent to accept a free HIV test, but our findings did not exclude the chance of no association. To the extent that urban day laborers in California have similar behaviors as rural farmworkers in North Carolina, predictors of intention to test for HIV within the following year included not having had a prior test and perceived risk for HIV infection, similar to our findings [19].

The findings of this exploratory study indicate that HIV is perceived as a serious problem in the farmworker community. Those that have been previously tested and have knowledge about HIV transmission were willing to use a home test kit. However, importantly, those that had the least knowledge and had not been tested did not have the same motivation to use a home test kit. A higher perceived risk for HIV in the community may be a prevention strategy, particularly if testing is offered in a residential setting. Opportunity to provide educational training on how to use a home test kit may engage those already knowledgeable as well as those that are not to avoid stigma associated with infection [24].

Before recommending the HIV self-test in this vulnerable population, healthcare and outreach workers should understand aspects of the validity and predictive value of the screening test. A recent meta-analysis of rapid HIV tests demonstrated that the oral fluid tests may be more appropriate in populations with a high prevalence of HIV [25], because the rapid test has a lower positive predictive value in low prevalence populations, which can result in more false positive screening results [26]. However, several studies showed that participants performed the home test correctly and were able to interpret the home test [27]. Further research is needed on training effectiveness, use of the test, and interpretation of results in a transient farmworker population. Additionally, more information is needed about the prevalence of HIV in seasonal and migrant farmworkers.

There are several limitations to this pilot study. First, women were not included, however, no MSF women were identified at the 19 camps on the days that we visited. The survey, which was not back-translated, consisted of 12 questions that was self-administered after the workday. We were unable to quantify the level of reading literacy for persons taking the survey, and some workers helped others with understanding the meaning of the questions. However, if a person could read, the survey was simple to understand. Participants self-selected themselves and there was an informal data collection process that allowed respondents to complete the survey in privacy but also opportunity for limited readers of Spanish to ask the outreach worker about the meaning of a question in a comfortable setting. While the outreach worker may have provided HIV testing and counseling to visiting MSF in the past, it is unlikely that this group of respondents received prior testing and counseling from the same outreach worker. Respondents were not routinely conversing with each other about the questions or their answers. For this reason, it is unlikely that results are due to information bias. Missing data may be problematic, but response rates for individual questions ranged from 93% to 98%, suggesting that missing data were distributed across individual questions. Residual confounding may be present; data were not collected on some key demographic and health behavior questions, such as history of STI, marital status, and number of sex partners. Cultural norms and values, frequency of cross-border migration, and contextual situations in the U.S. and their home countries could not be assessed, though would presumably impact a worker’s intention to choose a self-test. The strengths include a relatively large sample size, validated questions, and a high response rate. Further research is needed to replicate these findings in other settings and better characterize perceptions about HIV and validity of self-testing as a HIV screening approach in MSF.

## 5. Conclusions

Rates of new HIV infection in Latino men continues to increase in the U.S. While limited current data exist on HIV incidence rates or prevalence in this vulnerable occupational group, MSF, the annual movement of thousands of documented and undocumented male (and female) workers between the U.S., Mexico, and other countries engaged in agricultural production sets the context for HIV spread into the U.S., Mexico and beyond. Migrant and seasonal farmworkers residing in a rural area of eastern North Carolina expressed concern about HIV in their community, which deserves further investigation. This pilot study identified the potential acceptability to use HIV home test kits to increase HIV screening in MSF. Increasing awareness of HIV risk in the MSF by promoting self-testing may be an effective prevention strategy for this population Culturally-acceptable community-based initiatives may be warranted to include self-testing for HIV that could result in better access to testing for other STIs, which would improve the health status of farmworkers and their partners wherever they live.
